# Mini-implant placement under existing removable partial dentures decreased the mobility of remaining teeth in a randomized controlled 3-year clinical trial

**DOI:** 10.1007/s00784-025-06340-2

**Published:** 2025-04-24

**Authors:** Torsten Mundt, Ahmad Al Jaghsi, Friedhelm Heinemann, Christian Schwahn

**Affiliations:** 1 Department of Prosthodontics, Gerodontology and Dental Materials, University Medicine of Greifswald, Walther-Rathenau-Str. 42a, Greifswald, D-17475 Germany; 2 College of Dentistry, Clinical Sciences Department, Ajman University, Ajman, United Arab Emirates; 3 Center of Medical and Bio-Allied Health Sciences Research, Ajman University, Ajman, United Arab Emirates; 4 Private Practice, Morsbach-Lichtenberg, Germany

**Keywords:** Denture, Partial, Removable, Randomized controlled trial, Immediate dental implant loading, Mini-implant, Survival rate, Resonance frequency analysis

## Abstract

**Objectives:**

Longitudinal stability values of teeth after strategic implant insertion under existing removable partial dentures (RPD) are lacking. This explorative evaluation of a 3-year randomized controlled trial on strategic min-implants (MI) aims to estimate the stability changes of the remaining teeth and the tooth survival rates under different MI loading conditions.

**Materials and methods:**

Partially edentulous study participants of a university clinic and three private practices with inadequately supported RPDs received strategic MIs (diameter 1.8–2.4 mm). According to the randomization the MIs were either immediately loaded in group A or delayed loaded in group B. The longitudinal changes of Periotest values (PTV) for teeth were compared between groups using mixed models adjusted by sex, age, jaw, tooth site, center, and baseline PTVs. The tooth survival was estimated with Kaplan-Meier analyses and group differences were analyzed using Cox regression.

**Results:**

Altogether 232 MIs were placed under 48 mandibular and 31 maxillary RPDs with a total of 255 remaining teeth in both jaws. The initial median PTV of 9.5 in group A and 8.0 in group B decreased to 5.0 in group A and 2.0 in group B at the 3-year follow-up. In the fully adjusted model the tooth mobility reduction revealed 5.3 PTV units (95% CI: 3.5–7.2) in group A and 7.6 PTV units (95% CI: 5.4–9.9) in group B without inferiority of any group (*P* = 0.122). The 3-year tooth survival rates were 88% in group A versus 92% in group B without relevant group differences (*P* = 0.338).

**Conclusion:**

Strategic MIs under existing RPDs in persons with severe reduced dentitions decreased the mobility of the remaining teeth independent from implant loading modus. Further tooth loss can emerge despite of relieving the remaining dentition by the MIs.

**Statement of clinical relevance:**

Mini-implants as supplementary abutments can restabilize loose teeth in jaws with RPDs and unfavorable tooth distributions.

**Clinical trial registration:**

German Clinical Trials Register (Deutsches Register Klinischer Studien, DRKS-ID: DRKS00007589, www.germanctr.de), January 15th, 2015.

## Introduction

The authors of a recent comprehensive literature review with meta-analysis concluded that removable partial dentures (RPD) “typically did not lead to negative impacts on tooth loss or periodontal parameters”, if “professional re-evaluation and regular periodontal maintenance” are performed [1]. Without considering the remaining tooth number of the jaw, the calculated 5-year tooth survival rates of the meta-analyses were 95.1% for clasp-retained RPDs and 91.7% for double crown-retained RPDs. According to another review the tooth survival rates in moderately reduced dentitions (jaws with > 3 remaining teeth or shortened dental arches) ranged between 90% and 98% both for clasp-retained RPD and for double-crown retained RPDs after 3 to 5 years of observation [2]. However, the failure rate of double-crown abutments in severe reduced dentitions (≤ 3 remaining teeth) is markedly higher and ranged between 19% and 45% after 5 years [3–6]. Comparable data for clasp-retained dentures in severe reduced dentitions are not available. In a population-based study more than 50% of jaws with ≤ 3 remaining teeth were edentulous after 10 years of observation independently from the kind of denture [7]. It is to assume that in severe reduced dentition the abutments are often periodontally compromised or root-filled and might be overloaded by RPDs [3]. Unfavorable, i.e. unilateral distributions or few remaining teeth within the jaw increased the risk of failures of abutments and RPDs [5, 8–9].

One sign for periodontal damage or overloading is tooth loosening. Tooth mobility can be measured using clinical scoring systems or various electronic devices [10]. The most popular electronic device is the Periotest instrument [11]. Older evaluations with scoring systems showed no increase of the abutment tooth mobility after RPD insertion if regular recall visits and good oral hygiene are provided [12]. Newer studies with Periotest measurements of abutment teeth after RPD insertion showed either negligible changes up to 2 years [13–14], detrimental mobility increase in patients without a regular recall program after 10 years [15], or a medium-term tooth stabilization in clasp-retained distal extension RPDs [16] and in double crown-retained RPDs of severe reduced dentition [17].

If the number and/or distribution of abutment teeth are unfavorable, strategic dental implants can result in a more symmetrical support and stability of the RPD. In a systematic literature review of combined teeth and implant-retained RPDs, abutment tooth survival rates between 79.2% and 100% after 2 to 10 years of observation were reported [18]. In a similar meta-analysis of studies with tooth/implant supported double crown-retained RPDs the overall survival probability of abutment teeth was 93% (95% confidence interval [CI]: 85.4%-98.1%) after at least three years [19]. In a recent 5-year meta-analyses the abutment tooth survival rate was 92% for mixed attachments, i.e. ball attachments and/or double crowns and 95% for double crowns on teeth and implants [20]. Obvious supplementary implants might release the remaining natural abutments, longitudinal stability values of teeth for this treatment modality are lacking.

Minimal invasive and low-cost treatment alternatives for conventional two-piece implants are one-piece mini-implants (MI) with diameters below 3 mm. MIs were proved to be particularly suitable for the stabilization of complete dentures [21]. First prospective studies of MIs for the anterior support of mandibular free-end RPDs showed encouraging short- and middle-term results [22–26]. In a 3-year randomized controlled trial (RCT), strategic MIs were placed under existing RPDs and either immediately or delayed loaded between 2013 and 2018 [27]. The chewing efficiency [28] and patient`s satisfaction with the RPD [29] were faster improved after immediate than after delayed MI loading. There were no relevant differences between immediate and delayed loading regarding survival or stability of strategic MIs. However, the stability values for MIs were lower than for conventional implants and the MI failure rate in the maxilla was markedly higher than in the mandible [30].

The aims of the present explorative data analysis were to describe the longitudinal stability values for the remaining teeth and to estimate the tooth survival rates under different MI loading conditions in a risk model approach [31].

## Materials and methods

### Patients and treatments

**Fig. 1:**
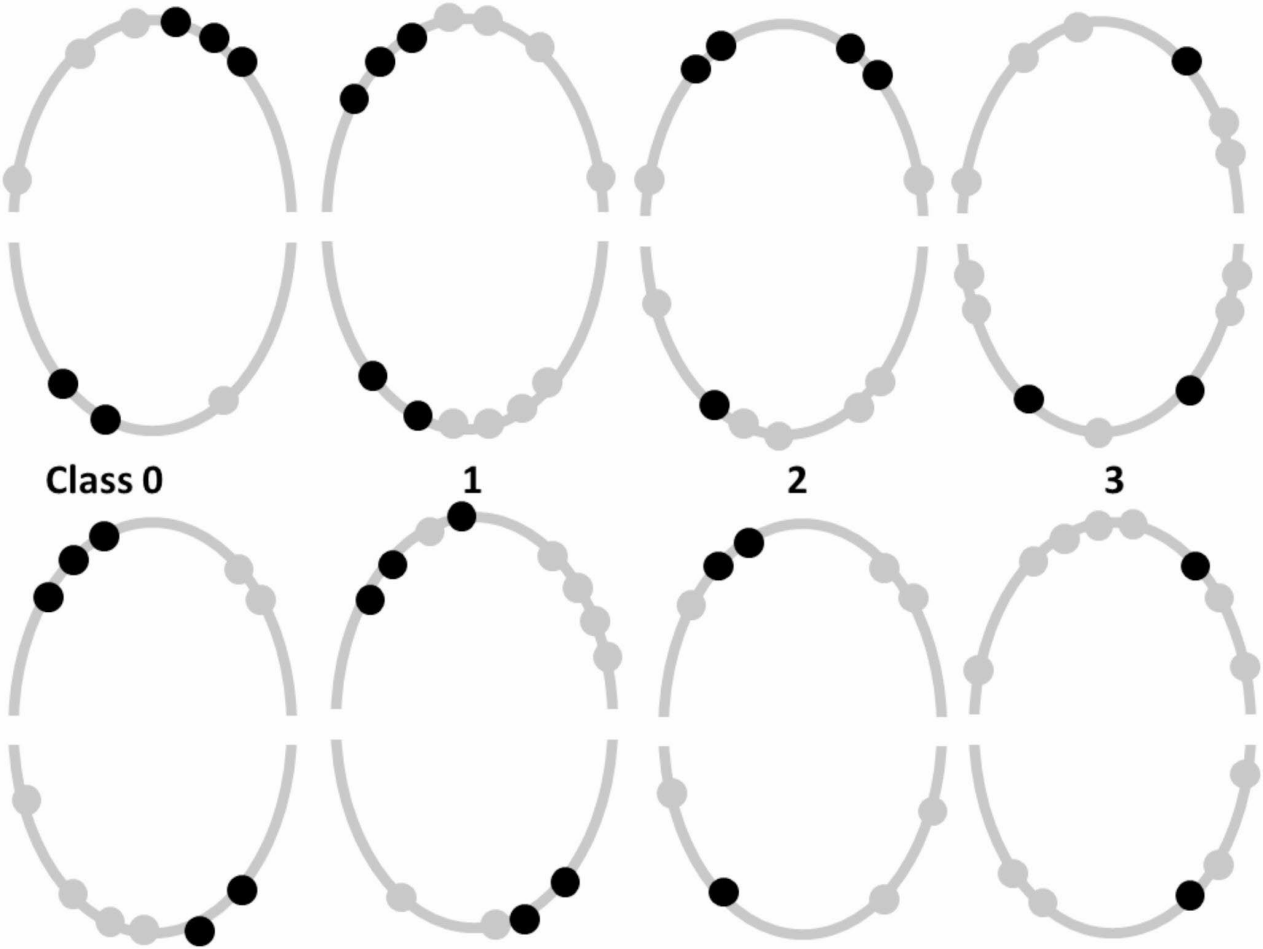
Tooth (grey) and mini-implant (black) combinations of the new classification on quadrant level for the maxilla (upper arch) and mandible (lower arch)

The report of this RCT with participants of one university clinic and three dental practices complies with the CONSORT-statement. The main inclusion criterion for a patient was an existing RPD with inadequate tooth support according to a new classification [27], i.e. either no (class 0), only incisors (Class 1), one (Class 2) or two (Class3) remaining posterior teeth and no canine in one or both quadrants of the study jaw (Fig. 1). The RPDs needed to be in good conditions albeit the patients were dissatisfied due to the inadequate support in the respective quadrants. The remaining teeth had to be in acceptable periodontal conditions (pocket depths ≤ 4 mm, no bleeding on probing, attachment loss < two third of the root length, mobility grade ≤ 2), vital or endodontically treated with a sealed root filling to the apical region without apical periodontitis [27]. The patients must be satisfied with the conditions of the non-study arch in which dental treatments were not required at the time of inclusion. For the primary endpoint bone level change at MIs a sample size of 26 participants per group was calculated.

Experienced dentists placed the MIs with ball attachments (Mini Dental Implant, MDI, Manufacturer in the past 3 M ESPE and now Condent, Germany) to the end that two abutments (teeth plus MI) per quadrant in the mandible or three abutments in the maxilla were in place (Fig. 1).

**Fig. 2:**
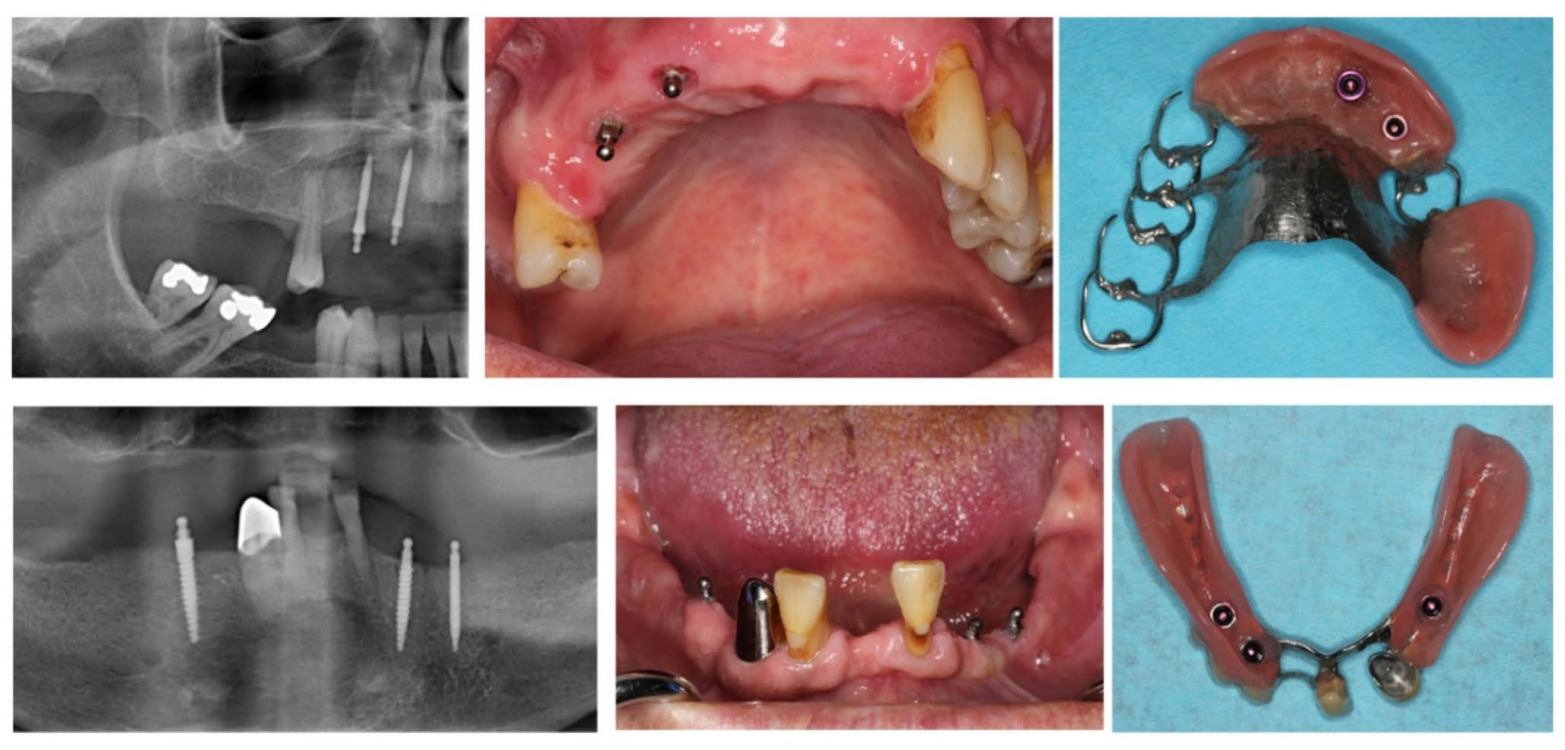
Examples of supplementary mini-implants for a 61 years old man with a clasp-retained maxillary denture and a 57 years old man with a double crown-retained denture

MIs with diameters of 1.8, 2.1, 2.4 mm and lengths of 10, 13, 15 mm were chosen according to the available bone, i.e. usually distributed in the region between maxillary sinus or mental foramina (Fig. 2).

After specific reduction of the denture base over the ball abutments the participants were allocated 1:1 to the loading groups. The statistician generated the random allocation sequence by center and jaw which was provided in a sealed envelope per participant. In the immediate loading group A, the matrices (housings with O-rings) were picked-up immediately if the insertion torque of all MIs were ≥ 35 Ncm. If the insertion torque of one or more MIs was < 35 Ncm, the RPDs of group A were soft relined. In group B the ball attachments remained unloaded. The final prosthetic connections of the soft relined RPDs in group A and all RPDs of group B were established after 4 months. Aftercare appointments were scheduled in combination with the follow-up examinations and performed afterwards. Replacements of abutments following tooth or implant loss after primary MI placement were not scheduled in the study protocol.

### Data assessment

The participants were clinically examined by one experienced and trained dentist before MI placement (T_− 1_), after two weeks (T_1_), 4 months (T_2_), 4.5 months (T_3,_ after housing pick-up in the patients with soft relined RPDs of group A and all patients of group B exclusively), one- (T_4_), two- (T_5_) and three-year (T_6_) after surgery. The day of tooth loss were acquired by the patient chart.

The tooth stability was measured using the Periotest instrument (Medizintechnik Gulden, Bensheim, Germany) [11]. A percussion rod of the instrument impacts at right angles to the tooth neck about 3 mm above the gingival margin 16 times for 4 s. The more stable the tooth, the quicker the percussion is. The instrument measures the contact time. This information is transformed to the Periotest value (PTV) on the scale between − 8 and + 50. PTVs are comparable with tooth mobility degrees of the Miller`s Index, i.e. degree 0 (physiological mobility 0.1–0.2 mm in horizontal direction): PTV range from − 8 to + 9; 1 (increased mobility to at the most 1 mm in horizontal direction): +10 to + 19, 2 (increased mobility exceeding 1 mm in horizontal direction): +20 to + 29, severe mobility both in horizontal and vertical direction: +30 to + 50 [11].

### Statistical analyses

PTVs are represented as median, first and third quartile because of asymmetric data distributions. Mixed models were used to compare the PTVs between groups at three levels, namely person, tooth site and time, based on the exact time points observed as a continuous variable [32]. Deviations from linearity for “time” were modelled by restricted cubic splines with three knots [33]. The group difference was adjusted by sex, age, jaw, tooth site, center, and by baseline PTVs as recommended [34]. Mixed models can handle intermediate missing values and use actual visit dates instead of planned visit dates, which respects the intention-to-treat principle and avoids the inferior “last observations carried forward” procedure [31]. Note that even missing values at the last examination at tooth level can be considered as intermediate missing values if an examination at patient level followed, as here to observe implants. In addition, robust HC1 variances were chosen to account for small deviations from model assumptions. The tolerance limit for non-inferiority of PTVs for any loading group was set at 2 units.

The Kaplan-Meier analyses for tooth loss based on the exact time points observed were performed as descriptive statistics assuming independent observations. However, the Cox regression considered dependence among teeth from the same patient by robust variances. Defined α-levels were not regarded and 95% CIs are primary presented to follow the recommendations of the American Statistical Association [35]. All statistical analyses were performed using Stata (Stata, Version 16.1; Stata Corporation, College Station, TX, USA).

## Results

### Characteristics of participants

**Fig. 3:**
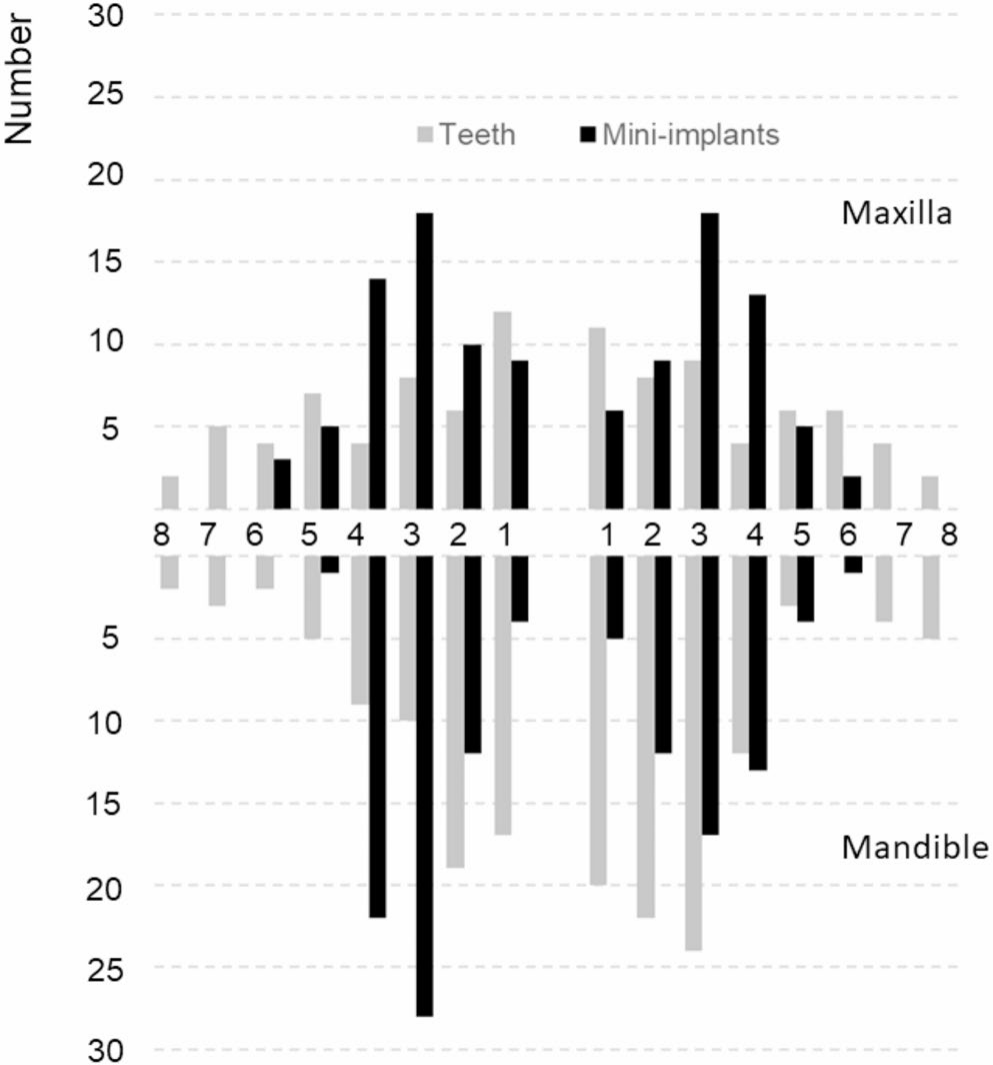
Baseline distribution for the 255 remaining teeth and 232 mini-implants of the study jaws by site according to the FDI tooth numbering system

From 88 initially assigned patients 76 participants received strategic implants according to the study protocol, among them three in both jaws. The new or optimized RPDs of the 79 study jaws (31 maxillas) were either double crown-retained (*n* = 52), retained by double crowns and clasps (*n* = 15), clasp-retained (*n* = 10), or precision attachment-retained (*n* = 2) and worn for at least two months. Figure 3 shows the distribution of all remaining teeth and MIs in the study arches just after surgery.

The total tooth number was 98 in the maxilla (median: 2.5, 1. quartile = 2; 3. quartile = 4) and 157 in the mandible (median: 2.5, 1. quartile = 2; 3. quartile = 5). Most of the study jaws showed in one quadrant no remaining teeth and only anterior teeth in one or both quadrants (Table 1).

**Table 1:** Number of study jaws by group and the new classification on quadrant level

Jaw classification	Number (%)	
	Group A	Group B
Class 0 (edentulous in one quadrant)	16 (40%)	19 (49%)
Class 1 (exclusively incisors in one or two quadrants)	15 (38%)	13 (33%)
Class 2 (one posterior tooth and no canine in one or two quadrants)	7 (18%)	5 (13%)
Class 3 (two posterior teeth and no canine in one or two quadrants)	2 (5%)	2 (5%)

Participants with study maxillas had RPDs (*n* = 19), fixed dental prostheses (*n* = 9) or natural dentition (*n* = 3) and participants with study mandibles showed complete dentures (*n* = 10), RPDs (*n* = 30) and fixed dental prostheses (*n* = 8) in their opposite jaw. A total of 112 maxillary (median 3 per jaw) and 120 mandibular (Median 2 per jaw) MIs were placed. Three participants received MIs in the maxilla and mandible. A total of 38 participants were allocated to group A (mean age 66.4 years, 22 women) and 38 participants were allocated to group B (mean age 65.4 years, 25 women). In group A, 34 RPDs of 32 participants were primarily soft relined because the insertion torque of at least one MI was < 35 Ncm and the MIs of 6 jaws were immediately loaded with housings. The losses to follow-up amounted to six participants in group A (16%) and seven participants in group B (18%), among them four in group A and five in group B after the second year.

### Tooth mobility

The initial mean and median PTVs for a total of 255 teeth in the study jaws for both groups together was more than halved after 3 years of observation (Table 2). The variation in the number of PTVs at follow-up was mainly due to intermediate missing values at tooth level, and to a lesser extent to the response rate of the participants to follow-up examinations, loss to follow-up after the first year, and tooth loss (Table 3). Out of group A six participants who immediately received the housing pick-up were only examined at the 4-month follow-up but not after 4.5 months.

**Table 2:** Unadjusted Periotest values for all remaining teeth of the study jaws over the 3-year period

Follow-up	Number	Mean	Standard deviation	1. Quartile	2. Median	3. Quartile
Baseline	246	10.7	8.85	5.0	9.0	15.0
2 weeks	227	10.4	8.97	4.0	9.0	14.5
4 months	215	10.6	9.59	3.9	8.0	15.0
4.5 months	194	9.7	8.99	3.5	7.8	12.5
1 year	197	7.3	7.70	1.9	5.2	10.0
2 years	166	6.4	6.97	2.0	5.0	9.0
3 years	138	4.4	6.18	1.0	3.9	7.0
Total	1383	8.9	8.67	3.0	7.0	12.0

**Fig. 4:**
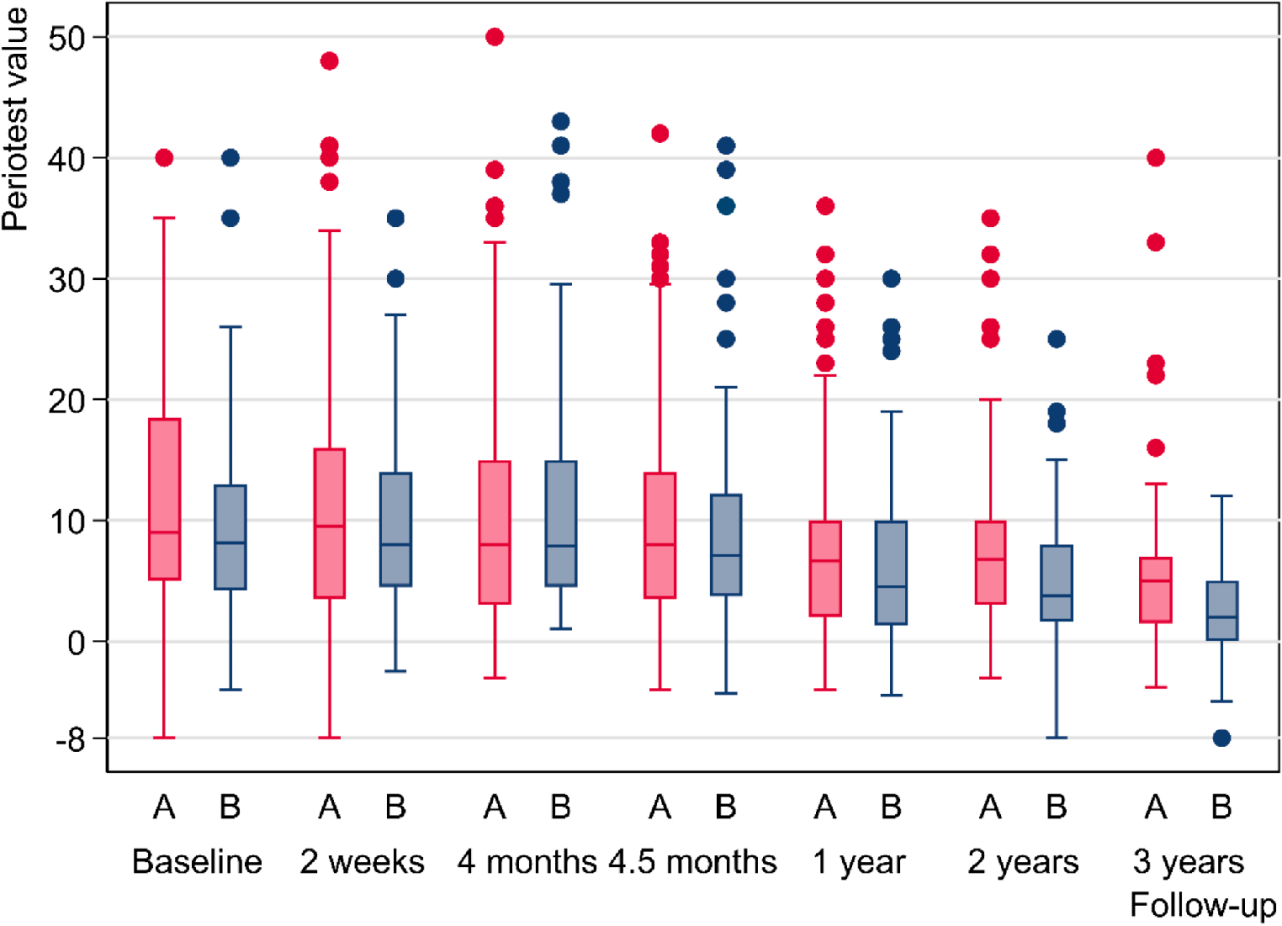
Unadjusted Periotest values of the teeth by group over the 3-year study period

For the raw data, the initial median PTV at 2 weeks after insertion was 9.5 for group A with immediate loaded MI and 8.0 for group B with delayed loaded MI (Fig. 4). As seen in the boxplot diagram the differences to the means can be explained by the upward outliers. The boxplots indicate a medium-term continuous decrease of the tooth mobility to the medians of 5.0 in group A and of 2.0 in group B at the 3-year follow-up.

**Fig. 5:**
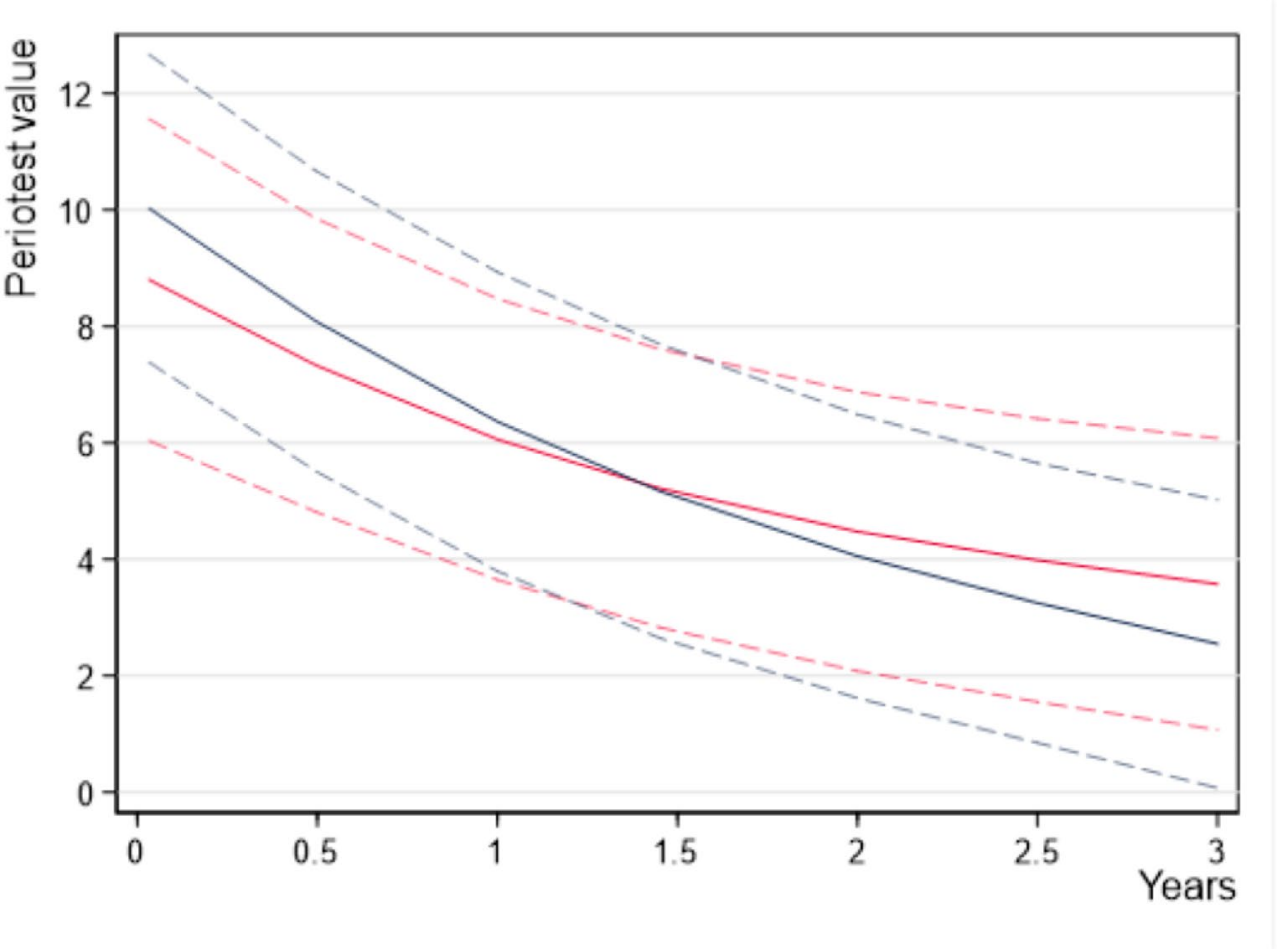
Adjusted Periotest values and the 95% confidence intervals (dashed lines) of group A (red lines) and group B (blue lines) over the 3-year study period

In the statistical modelling, PTVs with “time” as a continuous variable were adjusted by sex, age, jaw, tooth site, center, and baseline PTV. The absolute values in Fig. 5 refer to women, age of 65 years, mandible, the first premolar, the first study center and a baseline PTV of 10 (76 patients, 245 teeth in up to six follow-ups, 1109 observations).

Beginning with 8.9 in group A and 10.2 in group B, the estimated mean PVTs were 7.8 versus 8.8 after four months 6.1 versus 6.4 at one year, 4.5 versus 4.1 at two years and decreased up to 3.6 versus 2.6 at the 3-year follow-up. The contrast estimator of -1 after three years is less informative. The 95% CI after 3 years is out of the tolerance limit of ± 2 PTV units and is compatible with effects between − 2.9 in behalf of group B and 0.9 in behalf of group A (*P* = 0.299). The remaining teeth of group A show no inferiority compared with the teeth of group B. The tooth mobility decreased by 5.3 PTV units (95% CI: 3.5–7.2) in group A and by 7.6 PVT units (95% CI: 5.4–9.9) in group B. The contrast estimator for the decrease was 2.3 = 7.6–5.3 with a 95% CI of -0.6 to 5.2 (*P* = 0.122). As tooth loss might cause a bias in the tooth mobility in favor of survived teeth, the PTVs after one year are of interest. A total of 5 teeth were lost during the first year of observation. The PTV changes one year after MI placement were 2.8 (95% CI: 1.7 − 4.0) in group A versus 3.8 (95% CI: 2.6 − 5.0) in group B. The corresponding contrast estimator was 1.0 = 3.8–2.8 with the 95% CI of– 0.7–2.6 (*P* = 0.255).

### Tooth survival

**Fig. 6:**
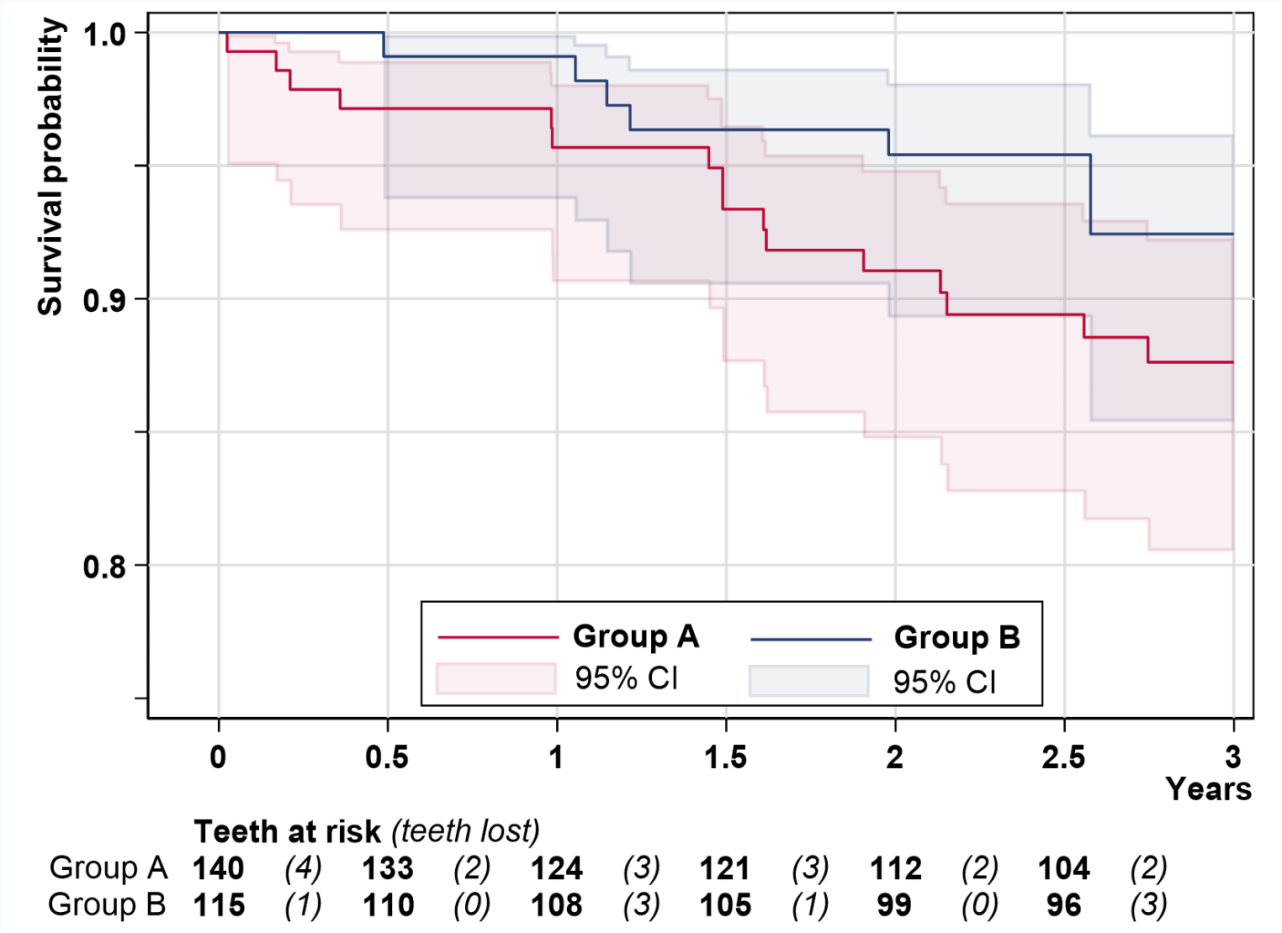
Kaplan-Meyer survival curves of the remaining teeth by group

Out of 145 existing teeth in group A and 115 teeth in group B at the time of MI placement, 16 teeth were removed among 10 participants in group A and 8 teeth were removed among 5 participants in group B during the three years (simultaneous tooth loss in the same patient: 2 teeth due to periodontal disease after 1.5 years in group A, 3 teeth due to fracture after 2.6 years in group B; Fig. 6). In total, 10 teeth were lost due to fracture (group A: 5), 11 teeth due to periodontal disease (group A: 9), and 3 teeth due to a combination of periodontal disease and caries (group A: 2). Additionally, one double crown abutment fractured and the root remained in group B. The extracted teeth (4 canines, 11 incisors, 9 posterior teeth) were 14 double crown abutments, 5 clasp abutments and 5 teeth without any retainer. Due to MI failures (12 MIs in five jaws [30]) and tooth loss, a total of 10 jaws met no longer the study design aim: to have at least three abutments per quadrant in the maxilla and two abutments in the mandible. No participant lost both MIs and teeth. At the final time point, there were 102 teeth available for 31 patients in group A and 93 teeth available for 29 patients in group B (Table 3). The cumulative tooth survival rate was 88% in group A and 92% in group B (Fig. 6). The Cox regression analysis considering depending observations within one participant shows a wide 95% CI of 0.5 to 6.4 for the relative difference of group A to B with the HR of 1.84 (*P* = 0.338).

**Table 3:** Corresponding number of patients and teeth at risk for each six-months period, as shown in Fig. 6

Year	Group A						Group B					
	Patients			Teeth			Patients			Teeth		
	At risk	Drop-outs (last observation in years; time point)	Patient became edentulous, but implants were still monitored (years; last tooth time point; last patient time point)	At risk (Fig. 6)	Teeth in drop-outs	Teeth lost (Fig. 6)	At risk	Drop-outs (last observation in years; time point)	Patient became edentulous, but implants were still monitored (years; last tooth time point; last patient time point)	At risk (Fig. 6)	Teeth in drop-outs	Teeth lost (Fig. 6)
0	38			140			38			115		
		1 (0.48; t_3_)			3			1 (0.39; t_3_)			4	
			0			4			0			1
0.5	37			133			37			110		
		1 (0.999; t_4_)			7			1 (0.65; t_3_)			2	
			0			2			0			0
1	36			124			36			108		
		0			0			0			0	
			1 (1.49; t_4_; t_5_)			3			0			3
1.5	35			121			36			105		
		1 (1.93; t_5_)			6			3 (2 × 1.93, 1 × 1.99; 3 x t_5_)			5	
			0			3			1 (1.98; t_5_; t_6_)			1
2	34			112			32			99		
		2 (1 × 2.01, 1 × 2.22; 2 x t_5_)			6			2 (2 × 2.05; 2 x t_5_)			3	
			0			2			0			0
2.5	32			104			30			96		
		0			0			0			0	
			1 (2.55; t_5_; t_6_)			2			1 (2.58; t_5_; t_6_)			3

t denotes the planned time point (t_3_: 4.5 months; t_4_: 12 months; t_5_: 24 months; t_6_: 36 months)

## Discussion

This explorative data analysis evaluated the mobility changes and the 3-year survival of the remaining teeth after immediate (Group A) or delayed loading (Group B) of strategic MIs for the RPD stabilization. Relevant group differences were not found albeit for the teeth of group B a trend to more reinforcement and to fewer losses compared to group A were found. The adjusted mean PTVs of the teeth decreased by more than half in group A and by about three fourth in group B over the observation period.

Some issues should be considered when interpreting the results. First, the primary outcome for the power estimation was marginal bone loss at MIs. Therefore, the present evaluations are rather explorative in nature and absence of evidence does not mean evidence of absence. Second, the response rate for all 76 participants over the whole study period was 83%, however, above the lower limit of 75% according to the protocol [27]. Moreover, 95% of the participants could be followed for at least 2 years. Third, the tooth number and distribution as well as retention elements of the RPDs in the study jaws were different. It is evident that patients with reduced dentitions “will always have a large variance of individual characteristics” both in the study and the opposite arch [36]. For that reason, only jaws with unfavorable support in one or both quadrants were included. It must be assumed that the load on the remaining teeth by the RPD should be fairly comparable before MI placement but an entire standardization is impossible. Subgroup analyses by jaw or by retention elements would be questionable due to the low number per group. Fourth, it could be that exclusively severe mobile teeth were lost and thus, the mean Periotest values decreased. This so-called survival bias is unlikely, as already after one year and five lost teeth the trend towards lower tooth mobility was proven.

The MIs used in this study have some advantages compared with standard-diameter implants. The screw-diameter of < 2.5 mm enables the use of less-complex surgical techniques such as bone spreading, splitting, planing or augmentations in areas with low bone thickness [21, 23, 26]. The low postoperative morbidity, the treatment effort and the moderate cost should move more patients to choose supplementary abutments for a better denture comfort [21, 23, 25–26]. There are also some disadvantages. MIs are well documented for overdenture stabilizations in edentulous mandibles [21, 23] with survival rates up to 100% after 10 years [37]. However, the number of middle or long-term studies in the edentulous maxilla and in partial edentulous jaws is still inadequate. The survival rate of MIs in the maxilla seems to be somewhat lower than in the mandible [21, 30, 38]. Divergences of MIs among each other and to teeth including dental retention elements can only be compensated within limits of about 20 degree contrary to double crowns on implants. The one-piece design of MIs can hinder an absolute no-load osseointegration. This fact could lead to complications in poor bone quality, e.g. in the maxilla [38]. In most of the MI systems, the implants have lengths of at least 10 mm. The available bone often hinders the placement in the molar region as seen in other reports [21, 38] and in this study. Augmentations of the maxillary sinus or the alveolar process for MIs are not performed. Therefore, a quadrangular support of free-end RPDs is to achieve only in few cases. Dentist and patients have to weight pros and cons in the shared decision-making process.

This is the first study that could demonstrate the positive impact of strategic implants on the mobility of the remaining teeth of jaws with severe reduced dentitions and RPDs. In most of the observational studies about supplementary abutments, implants and teeth were loaded simultaneously with the new denture contrary to our RCT [18–20]. In this way, the possible impact of strategic implants on tooth stability is not verifiable. In one prospective study of jaws with few remaining teeth [36, 39–40] and in some studies with free-end RPDs [41–43], standard-diameter implants were placed under existing RPDs similar to our study. However, changes in the tooth mobility were not evaluated by the researchers.

The mechanism of mobility reduction is plausible. Deflection and overload of the unilaterally or punctually located abutment teeth by retention elements, e.g. clasps and double crowns due to the caving-in of the mucosal supported contralateral, diagonal or free-end saddle could be diminished. Additionally, the occlusal forces on the dental arch including non-abutment and artificial teeth might be distributed more homogeneously following denture stabilization. This positive change of the RPD kinetics was mentioned in a number of reviews and studies [19–20, 39, 41, 42, 43]. In this context, the combination of various attachments, i.e. non-rigid ball attachments for implants and clasps or rigid double crows for teeth within one RPD is a matter of never-ending debates [18, 20, 40, 42]. Ball attachments for implants provide simple placement procedure even in limited interocclusal distances, low costs and, nonetheless, an effective retention [40, 42]. The housings with O-rings on the ball attachments of the present MIs provide only retention and no support for the prosthesis [44]. The prosthesis remains mucosal born in the MI region. More rigid attachments could afford a better RPD stabilization but also a higher implant loading. Recent developments are encouraging [45]. There is also the fact that particularly one-piece MIs are less expensive than two-piece conventional implants [21, 23]. Possible effects of different retention elements on the behavior of teeth, implants or prostheses can only be determined in RCTs [18, 20]. Differences in the tooth mobility changes between the groups in the present study were negligible. The trend to higher mobility decreases in the delayed loading group B compared with group A would be difficult of explanation. It has to be considered that the majority of the jaws in group A were treated according to the two-step immediate loading concept [46], i.e. initial soft relining and full loading by the housings four months later.

Similarly, the tendency toward a lower tooth loss rate after delayed loading than after immediate loading of the strategic MIs defy any conceivable explanation. Surprisingly, the number of necessary tooth extractions in the three-year period was relatively high contrary to our expectations. The survival rates of 88% in group A and 92% in group B were somewhat lower than the 3-year tooth survival rate of 95% (83–99%) of the latest meta-analysis of tooth- implant supported RPDs and mixed attachments. In this meta-analysis, the conventional implants and teeth were loaded simultaneously with a new denture except for one of eleven studies. In a study in which the conventional implants were also placed under 11 existing RPDs with severe reduced dentitions, the 6.5-year tooth survival rate was 89% [39]. In this study, only teeth with a periodontal attachment of more than 50% served as abutments and more than the half of the biological complication occurred after three years. In our study, an attachment loss up to two third was accepted. This could be one possible reason for the higher number of extractions within three years. A total 12 MI failures and 24 tooth losses led in only 10 jaws to inadequate abutment distributions according to our classification per quadrant. There was more than one MI failure in four participants and more than one tooth loss in five participants. Additionally, 11 incisors without any strategic importance failed. Whether some of the abutment losses had an impact on tooth mobility changes could not be statistically evaluated but may be expected. The replacement of strategic abutments by additionally MIs was performed after the 3-year study period. The tooth-specific periodontal prognosis is a significant predictor of tooth loss [47]. Noteworthy, all participants of the present study have experienced severe tooth loss before study inclusion. It is to assume that the behavioral, socio-economic and genetic factors for caries and periodontitis did not entirely disappear through motivation and continuous follow-ups including maintenance [48].

## Conclusions

Within the limitation of an explorative analysis the following conclusions can be drawn. Strategic MIs under existing RPDs in persons with severe reduced dentitions decreased the mobility of the remaining teeth independent from implant loading modus. Further tooth loss can emerge despite of relieving the remaining dentition by the MIs.

## Data Availability

Data are available on request from the corresponding author.
